# Supplementary material for: Efficient Frequency Conversion with Geometric Phase Control in Optical Metasurfaces
Source: Adv Sci (Weinh). 2022 Feb 20;9(12):2104508. doi: 10.1002/advs.202104508 (PMC9036049; doi:10.1002/advs.202104508)
Supplement: Supplementary file 1 — Supporting Information [file ADVS-9-2104508-s001.pdf]

Supporting Information

Efficient frequency conversion with geometric phase control in optical metasurfaces

Bernhard Reineke Matsudo[1], Basudeb Sain[1], Luca Carletti[2], Xue Zhang[3], Wenlong Gao[1], Costantino de Angelis[2], Lingling Huang[3], Thomas Zentgraf[1,]*

[1] Department of Physics, Paderborn University, Warburger Straße 100, 33098 Paderborn, Germany
[2] Department of Information Engineering and National Institute of Optics (CNR-INO), University of Brescia, Brescia 25123, Italy
[3] School of Optics and Photonics, Beijing Institute of Technology, 100081, Beijing, China

[*]Email: thomas.zentgraf@uni-paderborn.de

Contents

1. **Fitting procedure for the analytical model** **3**
2. **Nonlinear phase gradient design of the metasurface** **3**
3. **Influence of the Linewidth on the transmission and THG** **4**
4. **Nonlinear simulations in COMSOL** **4**
5. **Nanofabrication of metasurfaces** **5**
6. **Linear geometric phase response** **5**

1. Fitting procedure for the analytical model

The coupled anharmonic oscillator model described in the main text depends on various parameters, like resonance frequency and linewidths of both modes and the coupling constant between them. To analyze the experimental and numerical observations based on the model, we derived the model parameters for both cases. One way of deriving the model parameters utilizes the extinction spectra. The mathematical expression for the extinction spectrum derived from the coupled harmonic oscillator model can be presented as [1]:

$$\alpha(\omega) \sim \omega \, Im\left(\frac{\left(-\omega^2-\omega_d^2+2i\gamma_d\omega\right)^{-1}}{1-k^2\left(-\omega^2-\omega_d^2+2i\gamma_d\omega\right)^{-1}\left(-\omega^2-\omega_b^2+2i\gamma_b\omega\right)^{-1}}\right) \quad \text{(S1)}$$

Where ω is the frequency of the light and $\omega_{d/b}$ and $\gamma_{d/b}$are defined as the resonance frequencies and the linewidths of the Mie mode and the BICs, respectively. k represents the coupling constant.
On the other hand, the extinction spectra can be calculated from the experimentally and numerically obtained transmission spectra T as:

$$\alpha(\omega) = -\ln(T) \quad \text{(S2)}$$

Therefore, it is possible to transform the transmission spectrum into the corresponding extinction spectrum by using Equation S2, and the obtained spectrum can be used for fitting with Equation S1. The values for the coupling constant, the resonance frequency as well as linewidth of the Mie mode and the BICs can be extracted from the fit. The obtained parameters are shown in Table 1.

2. Nonlinear phase gradient design of the metasurface

As explained in the main text, the phase gradient is realized by identical nanoresonators, where each nanoresonator in a unit cell is rotated around its axis by an angle of α. Here, we increased the rotation from unit cell to unit cell along one direction by 22.5°. Therefore, a full rotation of 360° is covered by 16 unit cells and a PB phase shift of 4π (8π) in co-polarization (cross-polarization) is achieved. The corresponding deflection angle θ is obtained by applying the following grating equation [2]:

$$sin(\theta) = \frac{\lambda_{TH}}{2\pi}\frac{d\phi}{dx} + m\frac{\lambda_{TH}}{p} \quad \text{(S3)}$$

Where $\frac{d\phi}{dx}$ is the PB phase gradient due to the rotation of each nanoresonator, as explained in the main text and λ_{TH} is the TH wavelength of the generated light. One can obtain higher diffraction orders if the unit cell period p is larger than the third harmonic wavelength λ_{TH}. The different diffraction orders due to the unit cell are denoted by m. In our experiment we only observe the beam deflection due to the PB phase for a value of $m = 0$. Depending on the period, one expects diffraction orders at around 4.64° (co-polarization) and 9.29° (cross-polarization) for a TH wavelength of 430 nm.

3. Influence of the Linewidth on the transmission and THG

To link the numerical to the experimentally obtained results, we extracted the model parameters from the simulated transmission in Figure 1d and varied the linewidth γ_B of the BIC while keeping the remaining model parameters constant. We increased the linewidth γ_B from 0.1 nm to 6.5 nm, which is closer to the linewidth γ_B of the BIC observed in the fits to the experimental data (Table 1). Figure S1 shows the anharmonic model's linear transmission as fitted from the transmission values shown in Figure 1d for a linewidth of 0.1 nm and 6.5 nm. For a linewidth of 0.1 nm, the anharmonic oscillator model shows a transmission peak at 1320 nm, as expected from the numerical results. However, as the linewidth increases from 0.1 nm to 6.5 nm, the transmission peak's visibility is reduced, and the peak becomes a shoulder in the transmission dip of the mode x_D, which is similar to the experimentally obtained transmissions of the metasurface for different periods in Figure 3c. The decrease in visibility of the peak is related to a reduction of the nonlinear oscillator strength $x_{B,1}$, as shown in Figure S1. For a linewidth of 0.1 nm, Figure S1 shows a pronounced peak in the overall TH response related to THG from the BIC. However, as the linewidth is increased to 6.5 nm, the peak due to the BIC $x_{B,1}$, becomes insignificant, and the nonlinear response of the system whole system is dominated by $x_{D,1}$.

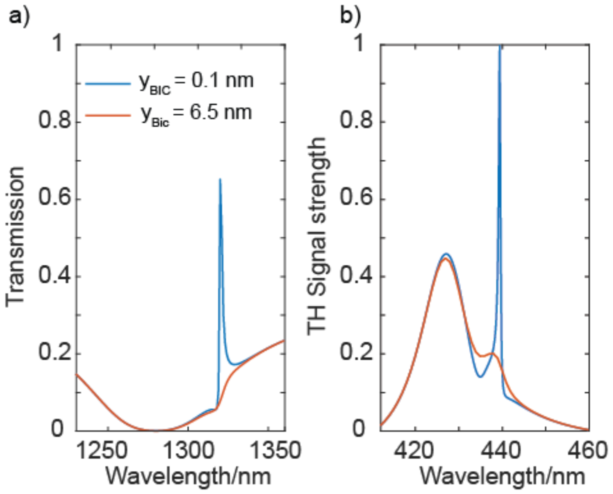

Figure S1. a) Linear transmission as calculated from the anharmonic oscillator model. We extracted the parameters for the model from the simulated transmission values in Figure 1d. However, the linewidth γ_B of the BIC was varied form 0.1 nm to 6.5 nm. C) the corresponding nonlinear oscillator strength for a linewidth of 0.1 nm and 6.5 nm.

4. Nonlinear simulations in COMSOL

We used a full-wave vectorial simulation in COMSOL to determine the third harmonic signal of the nanostructures in transmission. Periodic boundary conditions were applied along the x and y directions to simulate an infinite nanoresonator array. The simulations were carried out in two steps. First, we determined the spatial electromagnetic field distribuition of the nanoresonators at the fundamental wavelength, by using the finite element method in the frequency domain, Next, the TH field was calculated based on the nonlinear induced polarization which acts as a source for the TH response of the nanostructure. The nonlinear polarization is

determined by the electromagnetic field distribuition $E = (E_x, E_y, E_z)$ inside the nanoresonator and the nonlinear susceptibility tensor $\chi^{(3)}_{ijkl}$ of amorphous silicon, where the elements $i, j, k, l \in \{x, y, z\}$ denote to the cartesian coordinates. We considered the diagonal tensor elements $\chi^{(3)}_{xxxx} = \chi^{(3)}_{yyyy} = \chi^{(3)}_{zzzz} = 2.45 \text{ x}10^{-19} \frac{m^2}{V^2}$ and the off-diagonal elements were assumed to be $\chi^{(3)}_{iijj} = \chi^{(3)}_{ijij} = \chi^{(3)}_{ijji} = \frac{1}{3}\chi^{(3)}_{xxxx}$ with $(i \neq j)$. The complex refractive index of amorphous silicon utilized in the simulations was determined by ellipsometric measurements performed on an amorphous silicon film. The refractive index of silicon dioxide was set to 1.5.

5. Nanofabrication of metasurfaces

Metasurfaces were fabricated following a standard procedure of depositing amorphous silicon, patterning an etching mask with electron beam lithography (EBL), and inductively coupled reactive ion etching (ICP-RIE) of the silicon, along with subsequent removal of the mask. First, we deposited a 365 nm thin amorphous silicon film on a cleaned glass substrate by plasma-enhanced chemical vapor deposition (PECVD). A 130 nm thin film of PMMA resist was spin-coated on top of the deposited silicon film. The PMMA was baked out for 10 min at 180°C on a hot plate. Subsequently, the substrate was cooled down to 90°C on another hot plate for two minutes to avoid cracking the PMMA film. Afterward, a thin layer of the conductive polymer was spin-coated on top of the PMMA and baked out for 2 minutes at 90°C. The conductive polymer is used to avoid charging of the sample during EBL.

After spin-coating of the PMMA resist and the conducting polymer, an EBL process transferred the desired patterns into the resist. The conductive polymer was removed in water, and the PMMA was developed for 70 seconds in a 1:4 mixture of Methyl isobutyl ketone and Isopropanol. Afterward, the sample was immersed in isopropanol for 60 seconds and gently dried with nitrogen. After developing the resist, a 14 nm thick Cr layer was deposited on top of the resist by electron beam evaporation. In order to reveal the etching mask, the Cr film was lifted-off in hot acetone solution for one hour. Subsequent ultra-sonication removed the excess Cr completely. In the last fabrication step, the desired pattern was transferred from the Cr to the Si by ICP-RIE. Afterward, the Cr was removed in a commercially purchased Cr-etch solution.

6. Linear geometric phase response

At the fundamental frequency the phase response in the cross-polarization is given by the relationship:

$$\phi_{lin} = 2\sigma\alpha \qquad \text{(S4)}$$

Here, ϕ_{lin} is the linear geometric phase change in the cross polarization, α is the rotation angle of the nanostructure, and $\sigma = \pm 1$ defines the handedness of the circular polarization state. Figure S2 shows the phase response as a function of the rotation angle from 0° to 180° at a wavelength of 1325 nm for the cross polarization state. From

the figure it can be seen that the structure follows the behavior explained in equation S4. Note that only the cross-polarization carries the geometric phase in the linear domain.

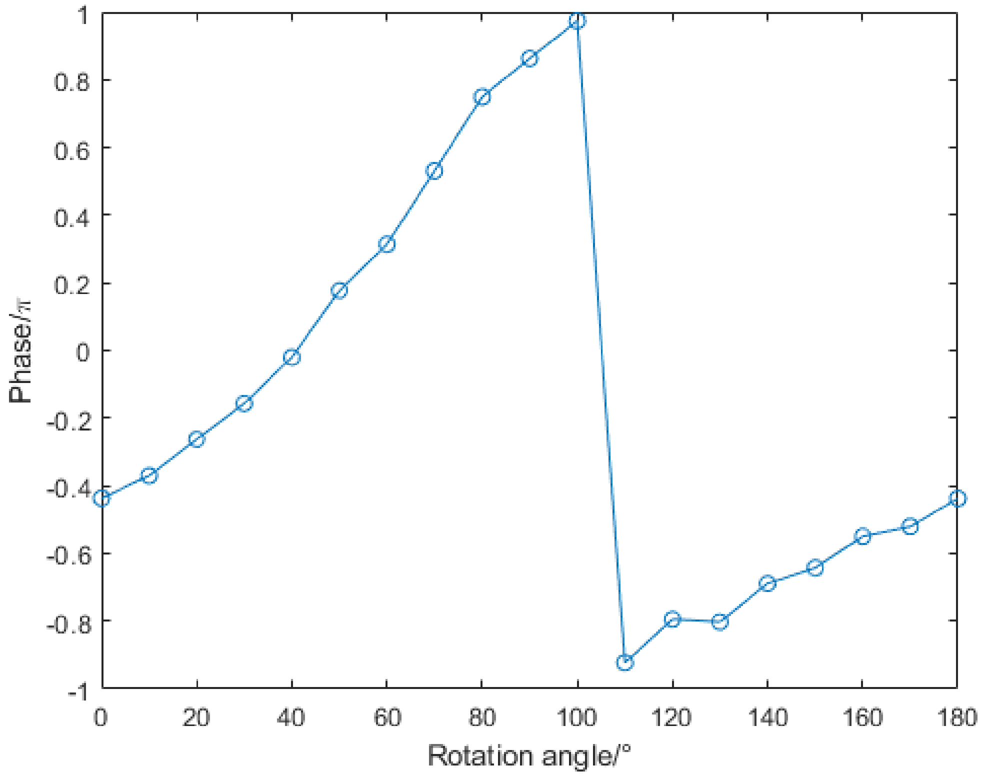

Figure S2. Linear geometric phase response of the metasurface at a wavelength of 1325 nm.

References

1. Metzger, B. Ultrafast nonlinear plasmonics : from dipole nanoantennas to hybrid complex plasmonic structures, 2014.
2. Eugene Hecht. Optik. In *Optik,* 7. Auflage; Hecht, E., Ed.; De Gruyter Studium; Walter de Gruyter GmbH: Berlin, Boston, 2018.
